# Protocol for *G*erman trial of *A*cyclovir and *c*orticosteroids in *H*erpes-simplex-virus-*e*ncephalitis (GACHE): a multicenter, multinational, randomized, double-blind, placebo-controlled German, Austrian and Dutch trial [ISRCTN45122933]

**DOI:** 10.1186/1471-2377-8-40

**Published:** 2008-10-29

**Authors:** Francisco Martinez-Torres, Sanjay Menon, Maria Pritsch, Norbert Victor, Ekkehart Jenetzky, Katrin Jensen, Eva Schielke, Erich Schmutzhard, Jan de Gans, Chin-Hee Chung, Steffen Luntz, Werner Hacke, Uta Meyding-Lamadé, the GACHE Investigators

**Affiliations:** 1 Department of Neurology, University of Heidelberg, Germany; 2 Department of Neurology, Krankenhaus Nordwest, Frankfurt, Germany; 3 Institute for Medical Biometry and Informatics, University of Heidelberg Medical School, Germany; 4 Department of Neurology, Vivantes Auguste-Viktoria-Klinikum, Berlin, Germany; 5 Department of Neurology, University of Innsbruck, Austria; 6 Department of Neurology, Academic Medical Center, University of Amsterdam, The Netherlands; 7 Department of Internal Medicine VI, Clinical Pharmacology and Pharmacoepidemiology, University of Heidelberg, Germany; 8 Coordination Center for Clinical Studies (KKS) Heidelberg, Germany; 9 Current Address: Department of Internal Medicine, Division of Infectious Diseases University of Texas Southwestern Medical Center at Dallas, U.S.A.Department of Neurology, University of Heidelberg, Germany

## Abstract

**Background:**

The treatment of Herpes-simplex-virus-encephalitis (HSVE) remains a major unsolved problem in Neurology. Current gold standard for therapy is acyclovir, a drug that inhibits viral replication. Despite antiviral treatment, mortality remains up to 15%, less than 20% of patients are able to go back to work, and the majority of patients suffer from severe disability. This is a discouraging, unsatisfactory situation for treating physicians, the disabled patients and their families, and constitutes an enormous burden to the public health services. The information obtained from experimental animal research and from recent retrospective clinical observations, indicates that a substantial benefit in outcome can be expected in patients with HSVE who are treated with adjuvant dexamethasone. But currently there is no available evidence to support the routine use of adjuvant corticosteroid treatment in HSVE. A randomized multicenter trial is the only useful instrument to address this question.

**Design:**

GACHE is a multicenter, randomized, double-blind, placebo-controlled, parallel group clinical trial of treatment with acyclovir and adjuvant dexamethasone, as compared with acyclovir and placebo in adults with HSVE. The statistical design will be that of a 3-stage-group sequential trial with potential sample size adaptation in the last stage.

**Conclusion:**

372 patients with proven HSVE (positive HSV-DNA-PCR), aged 18 up to 85 years; with focal neurological signs no longer than 5 days prior to admission, and who give informed consent will be recruited from Departments of Neurology of academic medical centers in Germany, Austria and The Netherlands. Sample size will potentially be extended after the second interim analysis up to a maximum of 450 patients.

**Trial Registration:**

Current Controlled Trials

ISRCTN45122933

## Background

### Scientific background

The treatment of herpes-simplex-virus-encephalitis (HSVE) remains one of the major unsolved problems in Neurology. The current gold standard in therapy is acyclovir, a drug that inhibits viral replication, but despite antiviral treatment mortality remains up to 15%. Even after early antiviral treatment, less than 20% of patients are able to go back to work and the majority of patients suffer from severe disability. Raschilas et al. [1] reported in 2002 a series of 85 patients in which 20% remained severely disabled, 28% moderately disabled, and only 14% of patients had a good recovery according to the Glasgow Outcome Scale (GOS). In a series of 34 patients a mortality of 12% was described, and 40% of the surviving patients presented an outcome ranging from moderate disability and severe disability to vegetative state [2]. This is a discouraging, unsatisfactory situation for treating physicians, the disabled patients and their families, and constitutes an enormous burden to the public health services. This has been an urgent topic during the last decade despite the availability of antiviral treatment with acyclovir.

Apart from direct virus-mediated tissue damage, secondary mechanisms have proven to play a significant role in the pathogenesis of HSVE [3-6]. Previous work has illustrated that the patients who suffer from HSVE present chronic progressive cranial magnetic resonance imaging (MRI) abnormalities despite early antiviral treatment with acyclovir [7]. These chronic progressive cranial MRI abnormalities have been also observed in an experimental mouse model of HSVE [8]. The mechanisms underlying this virus independent structural damage are thought to be autoimmune in nature [8]. The secondary mechanisms may involve the expression of immunologic NO synthase, matrix metalloproteinases and chemokines [4-6].

Viral load does not correlate with the severity of disease or with the extent of cranial MRI abnormalities in patients with HSVE [9]. In experimental models of HSVE the viral load is not influenced by treatment with corticosteroids. Despite the application of adjuvant corticosteroids, acyclovir effectively inhibits viral replication [10,11]. Corticosteroids given alone appear to have no adverse effect on the encephalitic process during experimental HSVE [12]. In experimental animal models of HSVE the adjuvant therapy with corticosteroids given together with the antiviral therapy has proven to be more effective than antiviral therapy alone in reducing the extent of structural abnormalities in brain tissue observed with MRI [11].

Before acyclovir was available, steroids were used regularly as the primary treatment for herpes-simplex-virus-encephalitis. Beneficial effects with this treatment were reported in large patient series [13,14]. Corticosteroids are also considered to be useful for the treatment of Herpes Encephalitis when they are administered together with the antiviral medication [3,15]. A recent retrospective analysis has reported a favorable outcome of patients with HSVE receiving adjuvant therapy with corticosteroids [15]. Adjuvant corticosteroids are also used for the treatment of patients with HSVE relapse [3].

The usefulness of an adjuvant corticosteroid treatment has been demonstrated for infectious diseases in which an exaggerated inflammatory response causes secondary deleterious effects which are independent from the infectious agent itself, such as during bacterial meningitis [16] and herpetical keratitis [17]. A recent prospective, multicenter, randomized, clinical trial has demonstrated the beneficial effect of adjuvant corticosteroids for the treatment of bacterial meningitis [16]. This study demonstrated that early treatment with dexamethasone improves the outcome in adults with acute bacterial meningitis and does not increase the risk of gastrointestinal bleeding.

Apart from this a recent Meta-analysis has demonstrated that short courses of high dose corticosteroids in patients with severe sepsis and septic shock have no deleterious effect on mortality [18]. This Meta-analysis also demonstrated that short courses of high dose corticosteroids do not increase the risk of superinfections, gastroduodenal bleeding or hyperglycaemia in patients with severe sepsis and septic shock [18].

The use of adjuvant topical corticosteroids during herpetical keratitis was controversial until a prospective, controlled trial was carried out, which demonstrated that a topical corticosteroid regimen was significantly better than no therapy in reducing persistence or progression of stromal inflammation and in shortening the duration of herpes simplex stromal keratitis [17].

Kamei et al. (2005) [15] have carried out a non-randomised retrospective study of 45 patients with HSVE. A poor outcome was evident with older age, lower GCS score at initiation of aciclovir, and no administration of corticosteroid. Older age, lower GCS at admission and no administration of corticosteroids were significant independent predictors of outcome.

### Rationale for the trial

The adjuvant use of corticosteroids has been recommended and used by experts from neurocritical care units in Europe and Japan for the treatment of herpes-encephalitis. So far however, this important question whether or not the adjuvant treatment with corticosteroids proves to be superior as compared to the standard antiviral treatment for HSVE, has not been addressed in a randomized trial.

The information obtained from experimental animal research and from recent retrospective clinical observations, indicates that a substantial benefit in outcome can be expected in patients with HSVE who are treated with adjuvant dexamethasone. But currently there is no available evidence to support the routine use of adjuvant corticosteroid treatment in HSVE. In our opinion, it is of utmost importance to address this question in a prospective, controlled multicenter clinical trial.

## Methods

### Study design and setting

The study design of GACHE is that of a multicenter, randomized, double-blind, placebo-controlled, parallel group clinical trial in adult patients with herpes-simplex-virus-encephalitis. The statistical design is that of a group sequential design with a maximum of three stages, rejection boundaries [19] and a potential sample size adjustment for the last stage [20]. Patients will be recruited from Departments of Neurology of academic medical centers in Germany, Austria and The Netherlands.

### Ethical considerations

Full ethical approval for this study has been obtained from all responsible Ethics Committees in Germany on August 28^th^, 2006 (ref: AFmu-106/2006). The principal investigator and/or his/her designee will obtain written informed consent from each subject or from the subject's legal representative or designee. Consent will be obtained before any protocol-specific procedures will be performed. All records will be kept confidential and the subject's name will not be released at any time other than the designee's or responsible government agencies.

### Study interventions

**Figure 1:**
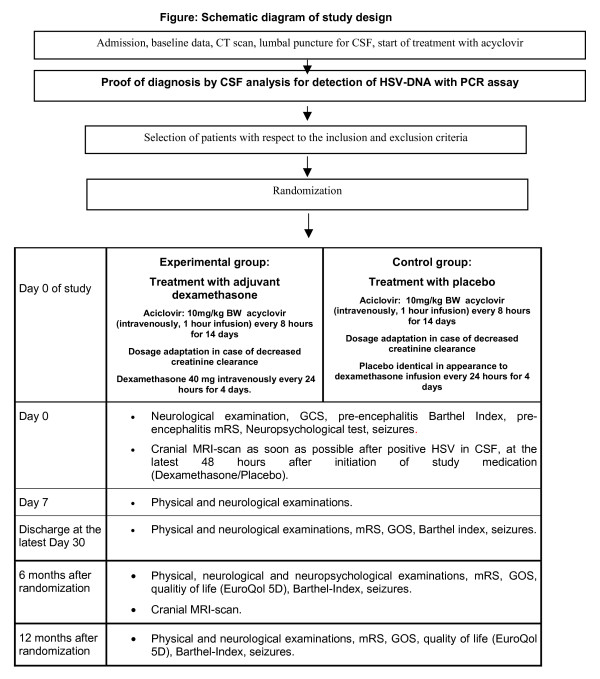
Schematic diagram of study design.

Patients will be randomly assigned to be treated either with acyclovir and dexamethasone or with acyclovir and placebo (Figure 1).

### Treatment with Acyclovir

Acyclovir is the standard antiviral drug for the treatment of herpes-encephalitis and is given for 14 days, as soon as medically indicated independent from time of randomization. Acyclovir is administered intravenously at a dosage of 10 mg/kg body weight (BW) every eight hours with an infusion time of 1 hour, if patients have a normal renal function. In case of reduced creatinine clearance (<60 ml/min) the acyclovir dosage will be adapted

### Study medication (dexamethasone/placebo)

Patients will be randomly assigned to receive study medication as soon as the diagnosis of herpes-simplex-encephalitis is confirmed with PCR. The study medication is administered intravenously: Dexamethasone at a dosage of 40 mg every 24 hours for four days, or placebo that is identical in appearance to the active drug.

### Labeling, dispensing, storage and accountability

Labelling of masked study medication (dexamethasone respectively placebo) and dispensing will be performed centrally by the Pharmacy of the University of Heidelberg. The study medication can be stored for three years at room temperature. A 'drug accountability log' or a corresponding document will be maintained at each trial center and has to be up-to-date at all times.

### Concomitant medication

All medications are allowed with exception of actual or long-standing corticosteroid treatment. Gastric protection with an antacid medication is obligatory during the administration of the study medication.

### Treatment compliance

The treatment procedures are administered in the trial centers during the acute phase of the disease; therefore there does not exist any problems with treatment compliance. The treating physicians as well as the patient are blinded.

### Primary and secondary endpoints

#### Primary endpoint

Binary functional outcome 6 months (± 14 days) after randomization (measured by the modified Rankin scale, a seven-point-scale from 0 to 6 points). A mRS of 3 to 6 will be seen as an unfavourable outcome (= failure). Patients dying between randomization and 6 months follow-up will be evaluated with mRS = 6.

#### Secondary endpoints

1. Mortality at 6 months after randomization.

2. Mortality at 12 months after randomization.

3. Functional outcome (Glasgow outcome scale: GOS) and quality of life (EuroQol 5D) 6 months (± 14 days) after randomization.

4. Functional outcome (mRS, GOS) and quality of life (EuroQol 5D) 12 months (± 28 days) after randomization.

5. Neuropsychological testing 6 months (± 14 days) after randomization.

6. MRI at 6 months (± 14 days) after randomization.

7. Seizures up to discharge (or at the latest at day 30), 6 months and 12 months after randomization.

### Selection and withdrawal of subjects

#### Subject inclusion criteria

• Age: ≥ 18 to ≤ 85 years.

• Laboratory – proven diagnosis of herpes-simplex-virus-encephalitis (PCR detection of HSV-DNA in the CSF)

• Focal neurological signs for not more than 5 days prior to admission.

• Informed consent.

• Women of childbearing potential: negative pregnancy testing in urine.

Every study center will keep a screening log which will contain the data of every patient who were initially treated with acyclovir due to suspected HSV-encephalitis during the enrolment period but who did not enter the study. Due to suspected diagnosis these patients could eventually be included in this trial.

#### Subject exclusion criteria

• History of hypersensitivity to corticosteroids.

• Systemic corticosteroid treatment within the last six months or at present time (>20 mg p.o. or generally intravenous intake).

• Two fixed dilated pupils.

• Pre-event score modified Rankin Scale (mRS) more than two or Barthel Index less than 95.

• Pregnancy.

• Breast feeding women.

• Recent history of active tuberculosis or systemic fungal infection.

• Recent head trauma/neurosurgery/peptic ulcer disease.

• Life expectancy less than three years.

• Other serious illness that confound treatment assessment.

• Simultaneous participation in another clinical trial.

• Previous participation in another clinical trial in the last 30 days.

• Previous participation in this clinical trial.

• Women of childbearing potential who are not using a highly effective birth control method.

• Acute viral infections other than HSVE (herpes zoster, poliomyelitis, chickenpox).

• Hepatitis B surface Antigen (HBsAg)-positive chronic active hepatitis.

• Approximately eight weeks before to two weeks after prophylactic vaccination.

• Lymphadenitis following Bacille Calmette Guérin (BCG) vaccination.

#### Subject withdrawal criteria

Patients will be withdrawn from the trial because of the following reasons:

• The patient or the legally authorized representative withdraws his/her declaration of consent or wishes to break off the trial.

• It becomes known after the patient's inclusion into the study that a major patient selection criterion was violated at the time of randomization.

The documentation of withdrawals will be done on a separate screening list of all patients.

A patient will be withdrawn from the treatment if one of the following events take place:

• When an unexpected event results in a medical treatment that is not compatible with the trial protocol.

• The undesired effect of a concomitant medication makes it necessary to discontinue the treatment according to the protocol.

• A severe adverse event necessitates the termination of the study treatment.

Patients withdrawn from treatment are not withdrawn from the study, but are followed and evaluated according to the protocol.

### Trial procedures for the individual patient

An overview over all examinations and tests carried out during the study is given in the following list:

Initial data on admission.- Baseline data: Age, sex, body weight and height, date and time of onset of symptoms, date and time of admission to the hospital, medical history, physical examination, GCS. CT scans on admission prior to randomization, seizures. Start of treatment

Day 0 (at randomization): Neurological examination, pre-encephalitis scores on mRS and Barthel-Index, GCS, Neuropsychological test. CSF analysis and result of positive polymerase chain reaction assay. Women of childbearing potential: birth control method, pregnancy testing in urine.

Day 0 or as soon as possible after positive HSV PCR, at the latest 48 hours after initiation of study medication (Dexamethasone/placebo): MRI-scan.

Day 7 to 10 after randomization: Physical and neurological examination.

At discharge, at the latest Day 30 after randomization: Physical and neurological examination, mRS, GOS, Barthel Index, seizures.

6 months after randomization: Physical and neurological examination, neuropsychological testing, mRS, GOS, Barthel-Index, cranial MRI, seizures, EuroQol-5D.

12 months after randomization: Physical and neurological examination, mRS, GOS, Barthel-Index, seizures, EuroQol-5D.

### Randomization

Study treatment allocation (dexamethasone respectively placebo) will be done in a ratio 1:1 by the method of minimization (Pocock and Simon 1975) considering two factors, study center and Glasgow coma scale (dichotomized: ≤ 8, >8). This method is preferred to a stratified block randomization because of the large number of strata (at least 50) which could possibly induce pronounced imbalance between the treatment groups. Each center will be provided with a series of study medication (dexamethasone or placebo) in a randomly order.

The described kind of randomization has to be done centrally. The preferred implementation is the web-based software **Randomizer **provided by the Institute of Medical Informatics, Statistics and Documentation of the Medical University of Graz  which ideally will be used by all participation centers. If this procedure is not not applicable for some centers, the procedure is carried out by persons of the IMBI, who are not involved in the data management or the statistical analysis of the study and who are called by phone. In special cases (on weekends), if it is impossible to receive the next allocation number in time (within two hours), the trial center has to use the next following code number of their preparation series. This has to be reported synchronously to the Institute of Medical Biometry and Informatics (IMBI). The next central allocation will minimize the possibly accrued imbalance.

### Blinding

GACHE is a double-blind clinical trial with the patient, the treating physician, the observer and all other site personnel as well as the monitor being unaware of the treatment assignment. Dexamethasone and placebo will be provided in identically appearing vials and study kits.

### Monitoring of adverse events

The independent DSMC will be responsible for reviewing subject safety during the trial. The major function of this committee will be to monitor the safety and efficacy of the study and to provide recommendations regarding further enrolment and conduct of the trial. The DSMC will periodically review tabulated safety summaries and additional safety data which the DSMC may request during the conduct of the trial. The DSMC is responsible for making recommendations to a Steering Committee regarding modifications or stopping of the trial based on observed safety and results of planned interim analyses.

### Statistics

#### Analysis sets

There will be three different analysis sets: the full analysis set, the per protocol analysis set and the safety analysis set. The full analysis set includes all patients who are randomized. The per protocol analysis set encloses all patients who were treated and observed as outlined in the protocol. The safety analysis set contains all patients who were treated at least for one day.

#### Analysis strategy

The primary endpoint analysis will be based on the full analysis set and will follow the intention-to-treat principle. For patients lost to follow up the last observation carried forward approach is applied. In addition sensitivity analyses will be added to estimate worst case and best case scenarios.

The statistical design will be based on a group sequential trial with a maximum of three stages and rejection boundaries according to O'Brien/Fleming [21]. After the second stage a sample size adjustment using the *conditional rejection probabilities approach *[22] and the sample size formula for binary data [23] is planned.

The (maximal) two interim analyses are carried out when one respectively two third of the originally planned patients (372 randomized patients; 312 patients without 15% drop outs) have passed the 6 months follow-up. In each of the analyses, the interim analyses and the final analysis, the nullhypothesis of equal failure rates p_control _(acyclovir monotherapy group) and p_exp _(group of acyclovir and corticosteroids) after 6 months

H_0_: p_control _= p_exp_   H_1_: p_control _≠ p_exp_

is tested by a (two-sided) χ^2^-test with one degree of freedom. The overall type I error rate is fixed to α = 0.05, the overall power to 1-β = 0.8.

If there is no significant result either in the first interim analysis or in the second interim analysis, a sample size adjustment for the last stage is carried out after the second stage.

In case of a significant result in any of the above described analyses, the same null-hypothesis of equal rates will be tested for the first secondary endpoint (mortality after 6 months), applying the same nominal significance level as in the test about the primary endpoint. Again, if this hypothesis can be rejected, the same null-hypothesis will be tested for the second secondary endpoint (mortality after 12 months), once more to the same nominal significance level. Due to the a priori ordering of the 3 hypotheses, this strategy can allow confirmatory conclusions for all tests without further increase of the overall type I error [24].

### Criteria for termination/extension of the trial

After each interim analysis the Data and Safety Monitoring Committee (DSMC) will review the data and the interim results and give recommendations to the Steering Committee (SC) whether to CONTINUE or DISCONTINUE the trial on the basis of the difference in the failure rates between the treatment groups and on the basis of the mortality rates:

a.) Failure rates after 6 months differ significantly between the groups:

The DSMC will recommend to STOP the study and the study will be STOPPED by the DSMC.

b.) Failure rates after 6 months do not differ significantly between the groups:

The DSMC will recommend to CONTINUE the study if there is no remarkable difference in the mortality rates between the treatment groups.

In addition, a sample size recalculation as described below will be carried out in the second interim analysis. The DSMC will recommend to CONTINUE the study until the newly estimated number of patients or a maximum of 450 patients are recruited.

The final decision to CONTINUE or to STOP the trial is made by the SC. The decision of the SC is based on the recommendation of the DSMC as well as important additional information available at the second interim analysis like new scientific knowledge, the actual patient enrolment and the financial situation.

### Detailed statistical methods

The detailed statistical methods (statistical tests, confidence intervals etc.) will be described in the statistical analysis plan for the interim and the final analyses the analysis plan for the final analysis. Here, especially treatment by center interactions (centers adequately pooled before unblinding) as well as treatment by GCS (Glasgow Coma Scale) interactions will be investigated in a logistic regression model.

### Sample size

In their large retrospective multicenter study of epidemiological features of herpes-simplex – encephalitis type I a failure rate of 35% for acyclovir monotherapy was reported [1]. The randomized clinical trial for dexamethasone in adults with bacterial meningitis [16] resulted in a failure rate of 25% in the placebo group and a failure rate of 15% in the experimental group. These results cannot be directly transferred because on the one hand the medical indication is different to GACHE and on the other hand in both cited studies the primary endpoint is based on the Glasgow Outcome Scale (GOS) and the time period of observation after randomization as well as the cut-off point for dichotomization is defined differently.

Due to this lack of data from former research about the distribution of the here chosen primary endpoint the study design of GACHE is based on a group sequential trial [19] with a maximum of three stages and an adaptation of the sample size in the last stage [22].

The sample size calculation is started with the pure group sequential design without taking into account the adaptation: For the planning phase at the beginning of the study a failure rate of p_control _= 0.4 in the acyclovir monotherapy group and a failure rate of p_exp _= 0.25 for the combination therapy of acyclovir plus corticosteroids is assumed. For a specified type I error rate of α = 0.05 and equally spaced stages this treatment difference can be detected with a power of 1-β_begin _= 0.8 if a maximum of 52 patients per group and stage are included, i.e. the maximum total sample size is 312 patients (ADDPLAN; Release 3). Assuming a rate of loss to follow-up of 15% 62 patients per group have to be enrolled in each stage which results in a maximum of 372 patients for the entire study.

In the second interim analysis a sample size recalculation based on the pooled data of the first and second stage is carried out and defines the final sample size for the entire study. First, the *conditional rejection probabilities *under the null-hypothesis H_0 _[22] are calculated. Using furthermore the estimated failure rates in the control group and in the experimental group for the variance estimation, a clinically relevant difference in the rates of 0.15 (as in the beginning) as well as a power of 90% for the last stage the sample size for the last stage is calculated [23]. This strategy, here called *combined strategy *of the groupsequential design and the adaptive design yields a final sample size of between 208 patients and (theoretically) infinity. To avoid a possible unrealistic large sample size a maximum limit for the total amount of patients has to be defined due to the rareness of the disease. At the moment as maximum limit a sample size of 450 patients for the whole study seems to be reasonable.

A simulation study for different failure rates in the control group (p_control _= 0.3; 0.4; 0.5; 0.6) and the fixed relevant treatment difference of 0.15 shows that the power of the described combined strategy is always slightly greater than the power of the pure group sequential design while maintaining the prespecified overall significance level of α = 0.05. The simulated power for the case of p_control _= 0.4 is 1-β = 0.82. As expected, in all simulated settings the average sample size (ASN) under the alternative H_1 _is also greater in the combined strategy than in the simple group sequential approach. However, if the assumptions upon the failure rate in the control group at the beginning of the study are not fulfilled the sample size adaptation after the second stage in the combined strategy leads to a smaller deviation from the desired power of 80% in comparison to O'Brien/Fleming [19]. Furthermore, the described combined strategy theoretically offers the possibility to change also other design aspects. However, if taken into consideration, such alterations always have to be considered with caution and thoroughly discussed by the DMSC and the SC. The one aspect, which can be of interest in this study, is the change of the maximum limit of 450 patients (which is based on practical considerations) depending on actual experience with the recruitment rate.

### Time plan for the GACHE Trial

Patient recruitment began in November 2007 and is planned to continue until June 2013. For the trial synopsis see Table 1.

**Table 1:** Trial synopsis

**Title of the Trial**	**German Trial of Acyclovir and Corticosteroids in Herpes-simplex-virus-Encephalitis (GACHE): a multicenter, multinational, randomized, double-blind, placebo-controlled German, Austrian and Dutch trial [ISRCTN45122933]**
Indication	Herpes-simplex-virus-encephalitis

Trial locations	Departments of Neurology of academic medical centers in Germany, Austria and the Netherlands.

Design	Multicenter, randomized, double-blind, placebo-controlled, parallel group clinical trial of treatment with acyclovir and adjuvant dexamethasone, as compared with acyclovir and placebo in adults with herpes-encephalitis. The statistical design will be that of a 3-stage-group sequential trial with potential sample size adaption in the last stage.

Objectives	The purpose of this study is to assess the efficacy and safety of adjuvant dexamethasone in the treatment of adult patients with herpes-encephalitis.

Eligibility criteria – inclusion	Proven herpes-encephalitis (positive HSV-DNA-PCR); age 18–85; focal neurological signs not longer than 5 days prior to admission, informed consent, women of childbearing potential: negative pregnancy testing in urine.

Eligibility criteria – exclusion	History of hypersensitivity to corticosteroids, systemic corticosteroid treatment within the last 6 months or at present time, two fixed dilated pupils, pre-event score mRS >2 or Barthel Index<95, pregnancy, breast feeding women, recent history of active tuberculosis or systemic fungal infection, recent head trauma/neurosurgery/peptic ulcer disease, life expectancy < 3 years, other serious illness that confound treatment assessment, simultaneous participation in another clinical trial, previous participation in another clinical trial in the last 30 days, previous participation in this clinical trial, women of childbearing potential who are not using a highly effective birth control method, acute viral infections other than HSVE (herpes zoster, poliomyelitis, chickenpox), Hepatitis B surface Antigen (HBsAg)-positive chronic active hepatitis, approximately eight weeks before to two weeks after prophylactic vaccination, lymphadenitis following Bacille Calmette Guérin (BCG) vaccination.

Treatment	Experimental Group: 10 mg/kg BW acyclovir (i.v., 1 hour infusion) every 8 hours for 14 days (Dosage adaptation in case of decreased creatinine clearance) + dexamethasone 40 mg intravenously every 24 hours for 4 days Control Group: 10 mg/kg BW acyclovir (i.v., 1 hour infusion) every 8 hours for 14 days (Dosage adaptation in case of decreased creatinine clearance) + Placebo every 24 hours for 4 days

Endpoints	Primary Endpoint: Binary functional outcome after 6 months measured by the modified Rankin scale (mRS), a seven-point-scale 0 – 6. A mRS-score of 3 to 6 will be seen as an unfavourable outcome. Secondary endpoints: Mortality after 6 and 12 months, functional outcome after 6 months measured by Glasgow outcome scale (GOS) and quality of life (EuroQol 5D), functional outcome after 12 months (mRS, GOS) and quality of life (EuroQol 5D), neuropsychological testing after 6 months, cranial MRI findings after 6 months, Seizures up to day of discharge or at the latest at day 30, and after 6 and 12 months.

Examinations/Follow-up	Day 0, 7, at discharge (at the latest at day 30), 6 months and 12 months after randomization.

Sample size	372 patients, potential sample size extension after the second interim analysis up to a maximum of 450 patients

Trial duration:	9 years: 2 years and 6 months preparation, 5 years and 6 months recruitment, 1 year follow-up.

Steering committee	Prof. Dr. Uta Meyding-Lamadé; Department of Neurology, Krankenhaus Nordwest, Frankfurt am Main Prof. Dr. W. Hacke, Director, Department of Neurology, University of Heidelberg Prof. Dr. N. Victor, Director, Institute of Medical Biometry and Informatics, University of Heidelberg

## Competing interests

As a conflict of interest it is necessary to mention that Merck provided the study medication (dexamethasone respectively placebo-IMP).

## Contributions of authors

UM-L, FM-T and WH were responsible for identifying the research question. UM-L, FM-T, WH, ESchi, ESchm, JdeG, MP, NV, EJ and KJ have all contributed to the development of the protocol and study design. FM-T, UM-L, SM, and MP were responsible for the drafting of this paper, although all authors provided comments on the drafts and have read and approved the final version.

## Appendix

The GACHE committees, national coordinators, clinical monitor, project manager, and investigators are as follows: **Steering committee**: W. Hacke (chair), N. Victor (cochair), U. Meyding-Lamadé (principal investigator); **Data Monitoring and Safety Committee**: U. Mansmann (Munich), D. Hanley (Baltimore), R. von Kummer (Dresden); **National coordinators**: Austria – E. Schmutzhard; Germany – U. Meyding-Lamadé; The Netherlands – J. de Gans; **Clinical Monitoring**: S. Luntz (Heidelberg); **Project Manager (since January 2008)**: S. Menon (Frankfurt); **Project Manager (2004–2007)**: F. Martinez-Torres (Heidelberg); **Investigators**: ***Austria ***– F. Gruber (Linz), B. Pfausler, E. Schmutzhard (Innsbruck), J Weber (Klagenfurt); ***Germany***: - K. Einhäupl, J. Bösel (Berlin, Charité); E. Schielke, M. Klein, G. Weissheit (Berlin, Vivantes); U. Schlegel M. Haupts H. Przuntek, T. Müller (Bochum); T. Klockgether, H. Urbach, A. Semmler (Bonn); D. Claus, R. Knoblich (Darmstadt); H. Reichmann, G. Gahn (Dresden); H.P. Hartung, B Hemmer (Düsseldorf); S. Schwab, J. Bardutzky (Erlangen); H.C. Diener, O. Kastrup (Essen); U. Meyding-Lamadé, V. Jost (Frankfurt am Main, Krankenhaus Nordwest); H. Steinmetz, T. Neumann-Haefelin (Frankfurt am Main); C. Weiller, S. Rauer (Freiburg); J. Liepert, C. Heesen, J. Gbadamosi (Hamburg); Francisco Martinez-Torres (Heidelberg); K. Fassbender, J. Osternage (Homburg an der Saar); A. Ferbert (Kassel); G. Deuschl, R. Stingele (Kiel); A. Wagner, D. Schneider (Leipzig); A. Grau, J. Wolf (Ludwigshafen am Rhein); T. Brandt, T. Rupprecht (München); Ringelstein W.R. Schäbitz (Münster); Hansen, N. Krause-Pape (Neumünster); R. Kaiser, M. Schellenschmitt (Pforzheim); U. Bogdahn, W. Jakob (Regensburg); M. Weller, A. Melms (Tübingen); Toyka, W. Müllges (Würzburg); ***The Netherlands***: - J. de Gans (Amsterdam).

## Pre-publication history

The pre-publication history for this paper can be accessed here:



## References

[B1] Raschilas F, Wolff M, Delatour F (2002). Outcome of and prognostic factors for herpes simplex virus encephalitis in adults: results of a multicenter study. Clin Infect Dis.

[B2] McGrath N, Anderson NE, Croxon NC, Powell KF (1997). Herpes simplex encephalitis treated with acyclovir: diagnosis and long term outcome. J Neurol Neurosurg Psychiatry.

[B3] Skoldenberg B, Aurelius E, Hjalmarsson A, Sabri F, Forsgren M, Andersson B, Linde A, Strannegard O, Studahl M, Hagberg L, Rosengren L (2006). Incidence and pathogenesis of clinical relapse after herpes simplex encephalitis in adults. J Neurol.

[B4] Meyding-Lamade U, Seyfer S, Haas J, Dvorak F, Kehm R, Lamade W, Hacke W, Wildemann B (2002). Experimental herpes simplex virus encephalitis: inhibition of the expression of inducible nitric oxide synthase in mouse brain tissue. Neuroscience Letters.

[B5] Martinez-Torres FJ, Wagner S, Haas J, Kehm R, Sellner J, Hacke W, Meyding-Lamade U (2004). Increased presence of matrix metalloproteinases 2 and 9 in short- and long-term experimental herpes simplex virus encephalitis. Neuroscience Letters.

[B6] Sellner J, Dvorak F, Zhou Y, Haas J, Kehm R, Wildemann B, Meyding-Lamade U (2005). Acute and long-term alteration of chemokine mRNA expression after anti-viral and anti-inflammatory treatment in herpes simplex virus encephalitis. Neuroscience Letters.

[B7] Meyding-Lamadé U, Lamadé W, Hess K, Degen O, Sartor K, Oberlinner CH, Hacke W (1999). Herpes simplex virus encephalitis: chronic progressive cerebral MRI changes in patients despite good clinical recovery. Clin Infect Dis.

[B8] Meyding-Lamadé U, Lamadé W, Kehm R, Heß T, Fäth A, Wildemann B, Haas J, Hacke W (1999). Herpes simplex virus encephalitis: chronic progressive cerebral MRI changes despite good clinical recovery and low viral load – an experimental mouse study. Europ J Neurol.

[B9] Wildemann B, Ehrhart K, Storch-Hagenlocher B, Meyding-Lamadé U, Steinvorth S, Hacke W, Haas J (1997). Quantitation of Herpes Simplex Virus Type 1 DNA in cells of cerebrospinal fluid of patients with herpes simplex virus encephalitis. Neurology.

[B10] Thompson KA, Blessing WW, Wesselingh SL (2000). Herpes simplex replication and dissemination is not increased by corticosteroid treatment in a rat model of focal Herpes encephalitis. J Neurovirol.

[B11] Meyding-Lamadé U, Oberlinner C, Rau P, Seyfer S, Heiland S, Sellner J, Wildemann B, Lamadé W (2003). Experimental herpes simplex virus encephalitis: a combination therapy of acyclovir and glucocorticoids reduces long-term magnetic resonance imaging abnormalities. J Neurovirol.

[B12] Baringer JR, Klassen T, Grumm F (1976). Experimental herpes simplex virus encephalitis. Effect of corticosteroids and pyrimidine nucleoside. Arch Neurol.

[B13] Habel AH, Brown JK (1972). Dexamethasone in herpes-simplex encephalitis. Lancet.

[B14] Upton AR, Foster JB, Barwick DD (1971). Dexamethasone treatment in herpes-simplex encephalitis. Lancet.

[B15] Kamei S, Sekizawa T, Shiota H, Mizutani T, Itoyama Y, Takasu T, Morishima T, Hirayanagi K (2005). Evaluation of combination therapy using aciclovir and corticosteroid in adult patients with herpes simplex virus encephalitis. J Neurol Neurosurg Psychiatry.

[B16] DeGans J, Beek D Van de, European Dexamethasone in Adulthood Bacterial Meningitis Study Investigators (2002). Dexamethasone in adults with bacterial meningitis. N Engl J Med.

[B17] Wilhelmus KR, Gee L, Hauck WW, Kurinij N, Dawson CR, Jones DB, Barron BA, Kaufman HE, Sugar J, Hyndiuk RA (1994). Herpetic Eye Disease Study. A controlled trial of topical corticosteroids for herpes simplex stromal keratitis. Ophthalmology.

[B18] Annane D, Bellissant E, Bollaert PE, Briegel J, Keh D, Kupfer Y (2004). Corticosteroids for severe sepsis and septic shock: a systematic review and meta-analysis. BMJ.

[B19] O'Brien PC, Fleming TR (1979). A multiple testing procedure for clinical trials. Biometrics.

[B20] Müller HH, Schäfer H (2001). Adaptive group sequential designs for clinical trials: Combining the advantages of adaptive and of classical group sequential approaches. Biometrics.

[B21] Jennison C, Turnbull BW (2000). "Group sequential methods with applications to clinical trials".

[B22] Müller HH, Schäfer H (2004). A general statistical principle for changing a design any time during the course of a trial. Statistics in Medicine.

[B23] Casagrande JT, Pike MC, Smith PG (1978). An improved approximative formula for calculating sample sizes for comparing two binomial distributions. Biometrics.

[B24] Maurer W, Hothorn LA, Lehmacher W (1995). Multiple comparisons in drug clinical trials and preclinical assays: a-priori ordered hypotheses. Biometrie in der chemisch-pharmazeutischen Industrie, J Vollmar (Hrsg), Fischer, Stuttgart.

